# Exploring nurse perspectives on AI-based shift scheduling for fairness, transparency, and work-life balance

**DOI:** 10.1186/s12912-025-03808-0

**Published:** 2025-09-02

**Authors:** Maisa Gerlach, Fabienne Josefine Renggli, Jannic Stefan Bieri, Murat Sariyar, Christoph Golz

**Affiliations:** 1 School of Health Professions, Bern University of Applied Sciences, Murtenstrasse 10, Bern, 3008 Switzerland; 2 School of Engineering and Computer Science, Bern University of Applied Sciences, Biel, Switzerland

**Keywords:** Work-life balance, Shift scheduling, Nurse retention, Artificial intelligence, Fairness, Transparency, Participation, Co-creation

## Abstract

**Introduction:**

Work-life balance (WLB) is critical to nurse retention and job satisfaction in healthcare. Traditional shift scheduling, characterised by inflexible hours and limited employee control, often leads to stress and perceptions of unfairness, contributing to high turnover rates. AI-based scheduling systems are promoted as a promising solution by enabling fairer and more transparent shift distribution. This study explored the perspectives of nurse leaders, permanent nurses, and temporary nurses on the perceived fairness, transparency, and impact on WLB of AI-based shift scheduling systems, which they had not yet used.

**Methods:**

A qualitative study design was used, with focus group (FG) interviews conducted between May and June 2024. FG interviews were conducted with 21 participants from acute hospitals, home care services, and nursing homes between May and June 2024. The interviews were analyzed using the knowledge mapping method, which allowed for a visual representation of key discussion points and highlighted consensus among participants. The discussions centered on five main themes: (1) experiences with current scheduling systems, (2) requirements for work scheduling, (3) fair and participatory work scheduling, (4) requirements for AI in work scheduling, and (5) perceived advantages and disadvantages of AI-based work scheduling.

**Results:**

Participants reported that current scheduling practices often lacked fairness and transparency, leading to dissatisfaction, particularly among permanent nurses. While temporary staff appreciated the flexibility in their schedules, permanent nurses expressed a desire for more autonomy and fairness in shift allocation. AI-based scheduling has the potential to improve shift equity by objectively managing shifts based on pre-defined criteria, thereby reducing bias and administrative burden. However, participants raised concerns about the depersonalisation of scheduling, emphasising the need for human oversight to consider the emotional and contextual factors that AI systems may overlook.

**Conclusion:**

AI-based scheduling systems were perceived as having the potential to be beneficial in improving fairness, transparency and WLB for nurses. However, the integration of these systems must be accompanied by careful consideration of the human element and ongoing collaboration with healthcare professionals to ensure that the technology is aligned with organisational needs. By striking a balance between AI-driven efficiency and human judgement, healthcare organisations can improve nurse satisfaction and retention, ultimately benefiting patient care and organisational efficiency.

**Supplementary Information:**

The online version contains supplementary material available at 10.1186/s12912-025-03808-0.

## Introduction

Good work-life balance (WLB) is critical for nurse retention and job satisfaction in healthcare [1]. WLB generally refers to an individual’s ability to successfully manage work and personal life demands, ensuring neither domain consistently dominates the other [2]. Lack of WLB is a leading cause of nurse attrition, with contributing factors including shift work, long hours, and rigid scheduling [3–5]. Shift scheduling plays a pivotal role in WLB as it dictates the structure and flexibility of nurses’ working hours [6, 7]. Research indicates that when nurses have limited control over their shift schedules, they experience increased stress and lower job satisfaction, often feeling a mismatch between professional obligations and personal needs, which can lead to perceptions of unfairness [8–10]. Furthermore, the scheduling practices for permanent nurses can be influenced by the interpersonal dynamics between employees and supervisors, with favoritism or strained relationships sometimes resulting in unfair workload distribution [11, 12].

Traditionally, shift scheduling in healthcare is managed using manual, self-managed, or rule-based digital systems. Manual scheduling relies on nurse leaders or administrative staff to assign shifts, which can be time-consuming and prone to bias, particularly when personal relationships influence decisions [13]. Self-managed scheduling, where nurses collaboratively create their own rosters, can enhance autonomy and improve WLB, but may lead to conflicts when preferences compete or unpopular shifts are systematically avoided [14]. Rule-based digital systems, which use predefined criteria to generate schedules, offer increased efficiency but lack the flexibility to adapt to unexpected changes, such as last-minute absences or evolving staff preferences. Although such systems can reduce administrative workload and generate schedules quickly, they are often unable to respond in real-time to individual staff needs or unplanned disruptions, limiting their effectiveness in dynamic clinical environments [15]. These conventional methods often struggle to balance individual preferences, fairness, and organizational demands, contributing to dissatisfaction and perceptions of injustice among nursing staff.

Incorporating nurses’ preferences into scheduling can enhance perceptions of organizational justice by accommodating individual requests for shifts and time off and by fairly distributing weekend shifts relative to employment levels [8, 13, 14]. This can be seen among nurses with temporary contracts, who often have greater autonomy over their schedules, leading to higher satisfaction with their WLB [16, 17].

To address these challenges, generative AI-based algorithms, which apply data-driven optimization and adaptability to allocate shifts according to predefined criteria, are promising solutions for creating fair and transparent shift schedules. Fairness is understood as the equitable distribution of shifts, considering employment level, personal needs, and workload balance [18]. Transparency refers to the visibility, understandability, and justifiability of how scheduling decisions are made, particularly whether the process and underlying criteria are clear to staff [19]. These systems can process large volumes of data by incorporating multiple factors, including unplanned absences and individual preferences, which are often too complex for manual scheduling [20, 21]. By making objective decisions based on predefined criteria, AI can reduce the risk of bias and ensure a more equitable distribution of shifts [20, 22, 23]. Unlike traditional scheduling methods, AI algorithms can continuously learn and adapt, offering dynamic and flexible scheduling that aligns with both staff needs and organizational demands [8, 23, 24].

In addition to promoting fairness and efficiency, AI-based scheduling systems have been shown to support nurses’ psychological wellbeing and reduce burnout, especially when they accommodate individual circumstances [25, 26]. Furthermore, AI implementation in nursing has been associated with increased productivity and time savings, provided that technological readiness and staff competencies are ensured [27]. Given these potential benefits, integrating AI-based scheduling tools in nursing could significantly enhance fairness and transparency in shift planning, thereby improving nurse satisfaction and retention [28–30].

Despite these advantages, implementing AI systems in healthcare remains ethically and practically complex. Ethical concerns of AI implementation in healthcare include the processing of sensitive personal data and the risk that biased training data, which seems also relevant for shift scheduling [31, 32]. Further concerns about transparency, autonomy, and the depersonalization of care highlight the importance of participatory development and ethical governance [33, 34]. Consequently, there is growing consensus that AI tools should be co-developed with nurses and nurse leaders to ensure that they reflect professional values and specific contextual needs [35]. In addition, digital competencies and leadership engagement are considered critical enablers for successful AI integration in nursing practice [8, 36, 37].

This ensures that the technology is usable, tailored to the needs of end users, and effectively addresses the challenges of shift scheduling in nursing [38].

To the best of our knowledge, and based on a comprehensive literature review conducted prior to this study, findings of which will be published separately, there is a notable gap in research on AI-based shift scheduling, particularly from the perspective of nurses, with an emphasis on fairness, transparency, and WLB. Given the distinct scope and depth of our literature review, it was not included as part of this paper to maintain a clear focus on the empirical findings.

Therefore, this study specifically aimed to explore the perspectives of nurse leaders, permanent nurses, and temporary nurses regarding fair, transparent, and participatory shift scheduling, as well as their perceptions of the advantages and disadvantages of AI-based scheduling.

## Method

### Design

To gain insight into the perspectives of nursing leaders and permanent and temporary nurses on fair and transparent AI-based scheduling, the authors utilised a qualitative research design by conducting focus group (FG) interviews between May and June 2024. A qualitative approach was chosen as it allows for an in-depth exploration of participants’ perceptions and expectations regarding shift scheduling practices, particularly in relation to fairness and transparency, as well as their perceived advantages and disadvantages of introducing AI-based systems for scheduling. Given that AI-based scheduling is an emerging topic in nursing, qualitative methods provide the flexibility needed to explore participants’ reasoning, concerns, and suggestions.

FG were selected to foster dynamic discussions, allowing participants to articulate and refine their views collectively. This format facilitated insights into group dynamics and the relational aspects of scheduling fairness that might not be revealed in individual interviews [39]. FGs provide an opportunity for participants to discuss their experiences in context, reflecting on organizational practices, power dynamics, and interpersonal influences that shape their perceptions of fairness. The real-time exchange of opinions enables the emergence of common themes and contrasting views, providing a richer and more nuanced understanding of how AI-driven scheduling is perceived across different nursing roles. Furthermore, the group setting stimulates recall and encourages collective sense-making, making it particularly effective for exploring unfamiliar or evolving topics, such as AI-based scheduling.

**Fig. 1:**
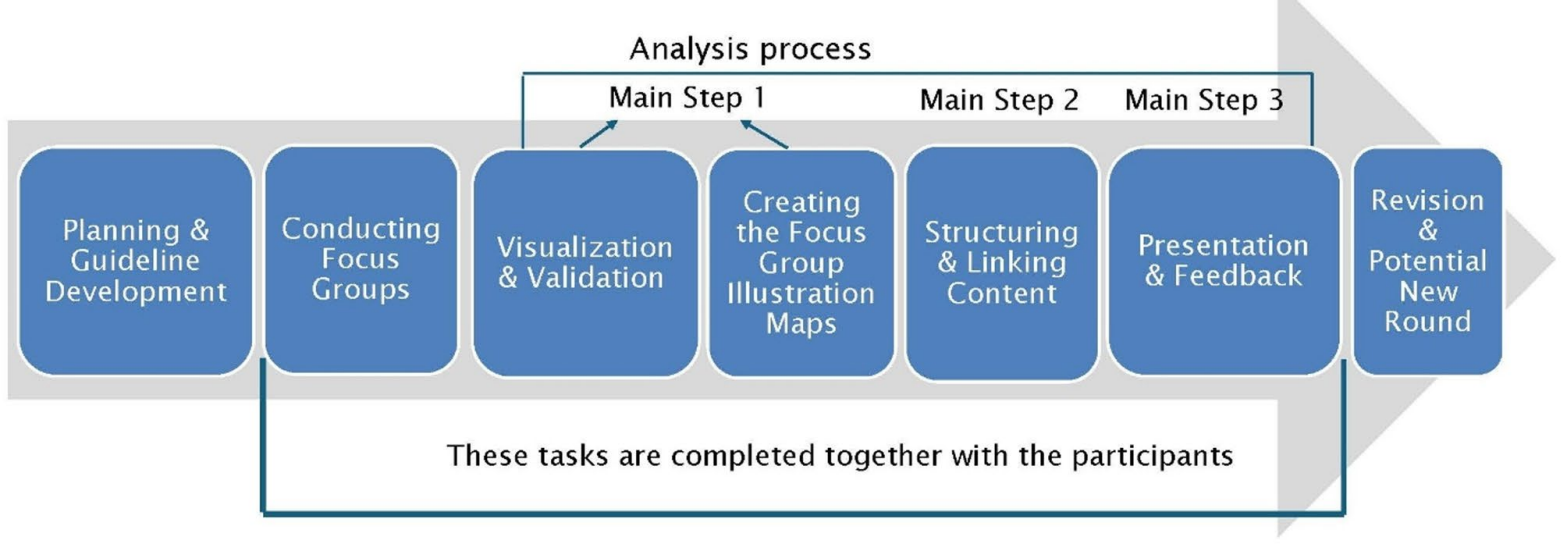
Knowledge mapping process

In this study, we applied the Knowledge Mapping method [40, 41] (Fig. 1). Knowledge Mapping was used both as a facilitation and a documentation tool during the FG. The process was iterative, involving three main steps: (1) real-time clustering of participant statements during the FG, (2) participant validation and refinement of clusters at the end of each session, and (3) consolidation and further refinement across FG to develop final thematic maps.

Although thematic analysis principles informed the Knowledge Mapping approach, it differs from traditional content analysis. No formal coding tree was developed; themes were clustered and visualized directly on Padlet boards based on participants’ inputs and validations. Padlet is a digital usability platform that allows users to create interactive boards and share content while developing mind maps.

This method enhances transparency in the analytical process, allowing participants to confirm or refine the identified themes, thereby strengthening the validity of the findings.

To ensure methodological rigor, the COREQ checklist (Consolidated Criteria for Reporting Qualitative Research) was used as the basis for describing the study [42].

### Study sample and recruitment

The authors employed a convenience sampling method to recruit permanent and temporary nurses, as well as nurse leaders, from diverse sectors, including acute hospitals, home care services, and nursing homes, in the German-speaking regions of Switzerland. This approach was chosen due to its pragmatic feasibility and ability to efficiently access participants with relevant professional backgrounds. Given the study’s focus on gathering a broad spectrum of perspectives, convenience sampling allowed for the inclusion of nurses with diverse roles and work settings, enabling a holistic understanding of the topic.

Specific inclusion and exclusion criteria were applied to ensure a diverse and relevant participant pool. Eligible participants were required to be registered nurses, nursing aides, or nursing leaders working in acute hospitals, home care services, or nursing homes, in either permanent or temporary positions. They needed to have at least two years of professional experience to ensure familiarity with shift scheduling practices and organizational structures. Additionally, fluency in German was essential, as the FG discussions were conducted in this language. Participants also had to be actively involved in or affected by shift scheduling in their workplace to ensure their perspectives were directly relevant to the study topic. Since AI-based shift scheduling is not yet implemented in practice, no experience with AI, particularly with AI-based shift scheduling, was required.

The study did not include professionals working in settings where no shift scheduling was required, such as outpatient clinics with fixed working hours.

The recruitment process took place between March and April 2024 and employed a multi-channel strategy. Potential participants were contacted through professional networks, including hospitals and home care services, as well as professional nursing associations. Recruitment materials were distributed internally within facilities and targeted emails were sent to eligible individuals. Interested participants received a flyer explaining the study’s aim, procedures, and participation details, and could register via email or phone. This ensured voluntary participation and allowed for clarification of questions before enrollment.

#### Context of the healthcare system in Switzerland

Switzerland has a decentralized healthcare system characterized by a mix of public and private service provision. Healthcare financing is primarily based on mandatory health insurance premiums, complemented by individual co-payments and public funding at the cantonal and municipal levels. While hospitals are largely planned and regulated by the cantons, privately organized institutions often provide ambulatory care.

The long-term and home care sector in Switzerland is diverse. Nursing homes are usually operated by public or non-profit organizations and provide residential care for older adults. So-called Spitex organizations deliver home care services. These include public, non-profit providers with a mandate to ensure access for all populations, and private, often specialized agencies. Spitex services range from basic nursing care and household assistance to complex medical treatments at home. Services are financed through a combination of health insurance contributions, public subsidies, and client participation.

To contextualize scheduling practices in the Swiss healthcare system, it is essential to note that full-time employees work 42 h per week, while the majority of nurses work part-time. A typical shift lasts an average of 8.24 h und the schedules should be provided four weeks in advance.

### Data collection

The CRSS method was employed in the formulation of the interview key questions. CRSS stands for “Collecting”, “Reviewing”, “Sorting”, and “Summarizing” [43]. Thus, the three interview guidelines for nurse leaders, permanent nurses, and temporary nurses were based on a structured review of relevant literature on AI-based shift scheduling, fairness, and nurses’ work-life balance (see Supplementary material 1–3). Each interview guideline consisted of open-ended questions addressing thematic areas such as scheduling experiences, perceptions of fairness, and expectations of AI. The research team reviewed the guides and informally piloted them to ensure clarity and relevance.

The identified themes were then translated into five main themes: (1) experiences with current scheduling, (2) importance of fair and participatory scheduling, (3) requirements for work-life balance, (4) expectations of AI-based scheduling, and (5) added value of AI. These themes formed the basis of the interview questions. Minor adjustments were made to the wording for the guides aimed at temporary nurses and nurse leaders. An overview of the themes and corresponding subthemes is provided in Table 1.

**Table 1:** Main themes of the interview guideline

Main themes of the interview guidelines	Question content
Experiencing current Work Scheduling	Satisfaction with the current Work Scheduling
	Control over Work Scheduling
	Impact of the work schedule on social environment/childcare
	Impact on work-life balance
	Challenges of certain shifts (8- and 12-hour shifts)
Requirements for Work Scheduling	Challenges and proposed solutions
	Satisfaction with the lead time of the Work Scheduling
	Flexibility in planning
	Control over Work scheduling
Fair and participatory	Understanding of fair Work Scheduling
	Challenges in dual roles (e.g. childcare)
	Favourite layers/layer patterns
	Factors influencing work-life balance
	Requirements for a good work-life balance
Requirements for AI in Work Scheduling	Confidence in AI-based Work Scheduling
	Influence on team dynamics
	Functions to improve the work-life balance
Advantages and Disadvantages of AI-Based Work Scheduling	Advantages
	Disadvantages
	Impact on work-life balance

The FG interviews were conducted online using Microsoft Teams [44] by the first and second authors, both female researchers with MSc degrees in Nursing Science and prior training and experience in qualitative methods research. At the time of the study, they were employed as research associates at a university of applied sciences in Switzerland. No prior relationship existed between the researchers and participants. The researchers introduced the study neutrally and encouraged open dialogue. All participants had their cameras and microphones turned on during the FG sessions, which enabled clear communication and supported active engagement. Each interview lasted between 40 and 90 min. No repeat interviews were conducted, and no non-participants were present. The decision to conduct the interviews online was based on both logistical and geographical considerations. Participants were recruited from different regions of German-speaking Switzerland, ensuring a diverse representation of nurses working in acute hospitals, home care services, and nursing homes. Conducting the FG online allowed for greater flexibility in scheduling and reduced the need for extensive travel, making participation more accessible for nurses with demanding shift schedules.

The study focused on German-speaking regions of Switzerland because German is the primary language of communication in the participating healthcare institutions. Additionally, limiting the sample to a single linguistic region ensured consistency in the discussion and avoided potential challenges related to language differences in qualitative data collection and analysis.

FG interviews were structured as facilitated discussions, with an additional project member assisting with moderation. This project member visualized participants’ statements in real time on Padlet, creating thematic clusters for each main theme. This interactive approach allowed participants to review and refine their contributions, fostering a more transparent and dynamic discussion process. In addition to the visualized summaries, the FG interviews were digitally recorded as a backup to address potential uncertainties in the visualization. However, the recordings were not analyzed and served only as a reference to ensure the accuracy of the documented discussions.

### Analysis

In line with the Knowledge Mapping approach [40, 41], analyses were carried out as an iterative process, beginning during the FG discussions. The initial step involved developing an interview guide that outlined the main themes of the study, based on existing literature and expert discussions within the research team. This guide formed the foundation for the FG discussions. The second step entailed conducting FG interviews using Microsoft Teams [40], which took place in German from May to June 2024. Each FG comprised 3 to 7 participants (see Table 1), ensuring a dynamic and interactive discussion. All participants completed the FG interviews. There were no withdrawals, and the data from all sessions were included in the analysis. Two researchers with backgrounds in nursing science and qualitative research moderated the interviews. Thematic consolidation and refinement were jointly carried out by two researchers, namely the first and second authors. A digital Padlet board was used in real time to capture participants’ statements and summarize key discussion points per main theme from the interview guideline. No additional qualitative data analysis software was used beyond Padlet. This visualization technique facilitated participant validation, ensuring their perspectives were accurately represented. At the end of each FG, participants reviewed and contributed to the credibility and reliability of the data. This real-time validation eliminated the need for returning full transcripts, ensuring accuracy without requiring post-session corrections. The third step of the analysis involved consolidating the Padlet boards from all FG into a single integrated thematic map. Sub-themes were derived inductively from participant contributions during and after the FG discussions. In this study, sub-themes were defined as broader conceptual patterns that emerged inductively from participant statements during the FG, allocated to a specific theme from the interview guide themes. While the interview guide provided a general structure for the discussions, the clustering of statements was data-driven and reflected participants’ own emphasis and framing of issues.

In line with principles of qualitative thematic analysis, the thematic clusters derived from the Knowledge Mapping process are conceptually closer to categories rather than fully developed theoretical themes as typically found in thematic analysis approaches. To enhance the trustworthiness of the findings, direct participant quotes were extracted from the audio recordings to illustrate key insights. The final sub-themes were organized around the five main themes outlined in the interview guidelines. Relationships between themes were visualized to highlight interconnections and underlying patterns. The sub-themes formed the basis for presenting results, ensuring the findings remained coherent and aligned with the research objectives. A formal coding tree was not developed; instead, sub-themes were clustered directly on the Padlet boards using inductive categorization (see Supplementary material 6). Data saturation was considered reached when no new sub-themes emerged in the later FG sessions.

## Results

A total of 22 nurses participated in four FG interviews. Most participants were female (95,5%, *n* = 21) and had an average age of 34 years (SD = 11.6). In addition, most of the participants worked 100% (68.2%, *n* = 15). In terms of care settings, most participants worked in an acute care hospital (31.8%, *n* = 7), followed by home care services (22.7%, *n* = 6), retirement, nursing homes (5.4%, *n* = 1); (22.7%, *n* = 5), leaders, and temporary staff (13.6% *n* = 3). Detailed information on participants’ characteristics is presented in Table 2. The responses are presented according to the five main themes summarized in Supplementary material 4.

**Table 2:** Participants charactaristics

FG	Setting	Participants	Number of distinct institutions
FG1	Acute care	Registered Nurses (5), Nursing Aides (2) (All permanent nurses)	Hospitals (4)
FG2	Home care + Retirement/Nursing homes	Registered Nurses (2), Nursing Aides (4) (All permanent nurses)	Home care organisations (2) Nursing homes (2)
FG3	Management (all settings) people who are involved in scheduling planning	Leaders (5), Commercial Assistant (1)	Hospitals (3) Home care organisations (1) Nursing homes (1)
FG4	Temporary working nurses (all settings)	Registered Nurses (3) (All temporary nurses)	Nursing homes, Hospitals (different)

### Experiencing current work scheduling

#### Organisational practices and challenges in work scheduling

Work scheduling in healthcare presents significant challenges for both leadership and staff, particularly in high-pressure environments such as intensive care units (ICUs). Nurse leaders described scheduling as a resource-intensive task that requires coordination across teams and departments. While ICUs often rely on fixed shift systems, general wards use standardized yet more flexible processes. Despite these differences, maintaining fairness in shift distribution remains a persistent challenge, especially in the face of fluctuating monthly workloads.

*Work scheduling is one of our most demanding tasks*,* particularly in intensive care units with fixed shift systems. Ensuring fairness remains a challenge…*,* …and in home care we rely heavily on staff flexibility*,* as service needs can change quickly. FG 3 (Nurse Leaders form hospital and Home care organisations)*.

#### Individual perspectives on work scheduling fairness and flexibility

Participants highlighted inconsistencies in scheduling practices and perceived gaps in fairness and transparency. In many institutions, long-term absences are managed manually, with shift lists circulated on paper for staff to sign up voluntarily. In contrast, short-term absences are often handled via informal communication, such as phone calls. While some organizations have implemented anonymous systems for last-minute adjustments, participants viewed these as insufficiently flexible or efficient, particularly in home care, where rapid staffing changes are common.

Across settings, staff noted that although fixed days off and leave requests were usually respected, two consecutive days off were not always granted, especially in private home care. This limited recovery time and contributed to dissatisfaction. Furthermore, while scheduling rules were formally the same for all employees, many participants perceived the planning process as opaque or inconsistent. One hospital attempted self-scheduling, but the approach did not increase satisfaction, as unpopular shifts were often still assigned to the same individuals.

The contrast between temporary and permanent staff also emerged clearly: temporary employees generally had greater flexibility in planning their schedules, while permanent staff had to adapt to fixed shifts. However, adaptability was still valued among temporary workers, as it increased scheduling chances and promoted collaboration. Two common contract types were mentioned: freelancers, who notify management of their availability, and floaters, who are assigned on short notice. Personal preferences were taken into account where possible.

### Requirements for work scheduling

#### Individual preferences and work-life balance needs

A growing demand for flexible, transparent, and equitable shift distribution has emerged in interviews with supervisors and employees. Prioritizing individual needs and fostering clear communication is essential for improving workplace satisfaction and efficiency. Participants expressed a preference for regular working days and shifts, which enhanced their WLB. Additionally, childcare considerations must be integrated into scheduling when employee preferences are considered. Options for 12-hour shifts should be available because some hospitals have successfully implemented models with both 12- and 8-hour shifts, particularly on weekends. This approach fosters patient continuity, while providing employees with greater flexibility. However, discussions continue regarding the potential drawbacks of 12-hour shifts, such as difficulties in maintaining concentration. Nevertheless, the participants welcomed the opportunity to choose their preferred shift model.

*Having regular shifts really helps with my WLB. What matters most is that our personal needs*,* like childcare or time off*,* are taken into account. FG 1(Registered Nurse*,* hospital)*.

#### Organizational flexibility and structural scheduling solutions

Employees in home care services desire the right to two consecutive days off, an equitable distribution of shifts (including night shifts), and consideration of personal preferences. Balanced WLB is essential, and overtime can be compensated for. At least two weekends per month should be free, and shifts should be adjustable to meet personal needs. Work schedules should be finalized approximately a month in advance.

*What we really need is two consecutive days off*,* that’s essential for recovery. Schedules should be planned at least a month ahead and take our personal situations into account. Flexibility makes a big difference in home care. FG 2 (Nursing Aides*, Home care organisation).

To promote a more adaptable work environment, initiatives should include flexible starting times, continuous pay, unpaid leave, and the ability to transition between departments. There should be clear rules for distributing weekends and other shifts.

### Fair and participatory work scheduling

#### Participatory planning and individual preferences

Engaging employees, acknowledging personal preferences, ensuring transparency, and facilitating regular communication enhances satisfaction and efficiency according to the participants. Consistent meetings and feedback mechanisms are vital to align work schedules with individual needs. A fair scheduling approach considers employee preferences and promotes an equitable distribution of early, late, night, and weekend shifts. Stability is prioritized by accommodating individual requests, minimizing shift changes, and allowing team members to swap shifts.

#### Equitable case and shift distribution in home care

Within the overarching theme of participatory work scheduling, nurse leaders in home care emphasized the importance of equitable distribution of both patient cases and shift times. They reported striving to balance the assignment of complex and less demanding cases to avoid overburdening individual staff members. To ensure fairness, the workload and start times should be distributed as evenly as possible among the team members. In some teams, staff preferences and feedback are taken into account when distributing assignments, reflecting a participatory approach adapted to the flexible structure of home care services.

### Requirements for AI in work scheduling

#### Functional requirements for AI-supported scheduling

The requirements for AI in work scheduling are particularly related to time savings and flexible accommodation compared with existing planning systems. AI-assisted scheduling should cater to diverse employee preferences and needs, including specific requests such as avoiding late shifts on certain days or temporarily working reduced hours (e.g., 40%) despite holding a higher contract (e.g., 50%) due to personal circumstances, to make up for the reduced time later. Additionally, the system must incorporate factors such as experience and educational levels, and account for supervisory roles and specialized responsibilities that extend beyond direct patient care. Effective management of fluctuating monthly and annual balances is essential, as is the efficient overtime administration. The system should also facilitate the tracking of student supervision days. Moreover, corrections and individual adjustments should be easily implemented, along with the ability to find replacements for short-term absences.

#### Strategic and team-oriented expectations of AI

AI should act as a neutral entity to promote team dynamics and be equipped with all the necessary information to enable optimal work scheduling. Therefore, a basic plan that can be corrected as needed is desirable. AI should be able to store work scheduling information from previous weeks and use it to distribute clients accordingly. All participants expected AI to save time for leaders, allowing them to focus more on their teams and patients rather than planning.

*AI needs to help us save time*,* that’s the biggest advantage. It should be able to consider individual preferences*,* like avoiding certain shifts or maintaining a reduced workload. At the same time*,* it has to take into account qualifications*,* leadership roles*,* and everything beyond bedside care. FG 3 (Nurse Leader*,* hospital)*.

### Perceived advantages and disadvantages of AI-based work scheduling

The anticipated implementation of AI-based work scheduling is perceived to offer significant potential benefits across various healthcare sectors, including acute hospitals, home care services, and nursing homes. Participants expected advantages such as higher planning accuracy and efficiency, a reduction in planning errors and administrative burden, and a fairer distribution of shifts. These optimizations were perceived as potentially leading to more time available for patient care, higher employee satisfaction, and improved working conditions characterized by greater transparency and fairness.

However, participants also anticipated several challenges associated with the use of AI, including increased dependence on technology and the loss of human elements, such as emotional sensitivity and potential acceptance issues among employees.

#### Perceived advantages

Participants described a range of expected benefits associated with AI-supported work scheduling. They anticipated that AI could enable more precise and efficient planning, potentially improving the quality of work schedules. Owing to its ability to make changes and adjustments quickly and easily, participants expected high flexibility in the scheduling process. They believed that task and shift assignments could be made more effectively based on employees’ qualifications and experiences, thereby increasing overall efficiency.

Another major advantage perceived was substantial time savings and a reduction of errors in the planning process through the use of AI.

Furthermore, participants expected that AI could act as a neutral entity, reducing the emotional burden on individuals responsible for planning. Decision-making based on objective data and algorithms was anticipated to increase the objectivity of scheduling and positively influence team dynamics.

*If AI can really plan based on our qualifications and experience*,* that would make things much more efficient. It also helps when decisions don’t feel personal anymore*,* it takes the pressure off the people who usually have to make those tough calls. FG 1(Registered Nurse*,* hospital)*.

#### Perceived disadvantages and expected limitations

At the same time, participants expressed concerns about the expected limitations. They assumed that fully accommodating all individual needs and preferences would remain unlikely, and discrepancies would persist. The ability to consider individual requests might be restricted, thus limiting personalization.

Participants also anticipated a high dependency on the technical system, which could be problematic particularly in the case of technical failures. They noted that the system’s effectiveness would depend heavily on the quality and currency of the input data.

For leaders, the potential loss of human aspects in planning was seen as a disadvantage. Participants highlighted that the current emotional or physical burden on employees might not be adequately reflected by AI. Human intuition and sensitivity to individual needs were viewed as elements that AI could not fully replace.

Despite the use of AI, participants emphasized that responsibility for scheduling should remain with human planners. It was considered essential that humans retain final control over work schedules to ensure they meet department-specific requirements and human needs. Furthermore, participants underlined the necessity for AI systems to be designed and trained to uphold principles of fair and effective work scheduling.

*Of course*,* no system will ever be able to meet everyone’s preferences completely*,* there will always be differences. And if the data isn’t correct or up to date*,* the whole thing doesn’t work. We also depend a lot on the system*,* which can be a problem when there’s a technical issue. In the end*,* I think it’s important that people still have the final say. A computer can’t feel how someone’s really doing. FG 2 (Registered Nurse*,* home care organization)*.

## Discussion

The findings of this study provide a nuanced understanding of the perspectives of nurse leaders, permanent nurses, and temporary nurses regarding fair, transparent, and participatory shift scheduling.

The results indicate that while both nurse leaders and nurses emphasize the importance of fairness, transparency, and flexibility in work scheduling, their priorities differ. Nurse leaders primarily focus on operational efficiency, workload management, and the strategic use of AI to streamline scheduling processes. In contrast, nurses emphasize the impact of scheduling on WLB, recovery, and individual needs. Regarding AI, nurse leaders value its potential for neutral and objective planning but cautions against the loss of human sensitivity. Nurses appreciate AI’s fairness but stress the necessity of human oversight to accommodate personal and emotional factors. Both groups agree that ultimate scheduling responsibility must remain with human planners.

Moreover the participants’ statements emphasise the research on of work scheduling on WLB, job satisfaction, and nurse retention. Participants highlighted the need for flexibility, regular working days, and consideration of personal preferences, including childcare, and the ability to engage in personal activities. These aspects are crucial for improving WLB and reducing burnout. Notably, studies have shown that inflexible and inconsistent scheduling contributes to high turnover rates [45]. This is further supported by recent evidence demonstrating that AI-supported planning systems can enhance nurses’ WLB by aligning schedules with personal needs, thereby reducing stress and increasing job satisfaction [25].

The notion of fairness in scheduling also came under scrutiny. While AI can create equitable schedules based on predefined rules, questions remain about whether these rules themselves are fair. For instance, allowing employees to work only on specific days, such as Mondays and Tuesdays, can be seen as both fair and unfair, depending on the broader context and organizational needs. These subtleties highlight the importance of involving staff in defining what fairness means within their specific setting, rather than imposing a one-size-fits-all solution [8, 46]. Therefore, it is essential to use context-specific training data. For instance, training an AI model with hospital data does not ensure fairness in home care services or nursing homes. A growing body of research suggests that ethical AI development requires the active participation of nurses to ensure the system aligns with professional and organisational values [35].

AI-based work scheduling offers several advantages that address the challenges identified in the traditional scheduling methods. Participants emphasized the increased planning accuracy, efficiency, and fairness associated with AI, which aligns with the literature on AI’s potential to enhance operational efficiency and reduce errors [47]. AI’s capacity to handle large datasets and make objective decisions can minimize biases and improve perceptions of fairness, supporting the finding that AI promotes more equitable scheduling [48]. The time-saving potential of AI for nurse leaders, enabling a greater focus on patient care and leadership tasks, was also highly valued. These findings are in line with recent studies showing that open-source AI scheduling systems can efficiently account for personal preferences and institutional rules, generating high-quality rosters with minimal manual adjustment [15].

Despite these advantages, the participants expressed concerns about the potential loss of human elements in planning. AI may not fully capture the nuanced understanding of individual employee needs and current workloads that human planners can provide. This concern is echoed in the literature, where the depersonalization and rigidity of AI systems have been noted as significant drawbacks [49]. The participants emphasised the importance of maintaining human oversight to ensure that AI decisions are aligned with departmental needs and values. Furthermore, they highlighted the necessity for a balanced approach that combines AI efficiency with human intuition. In planning, the human factor is of particular importance, as it encompasses both decision-making power and an empathetic understanding of individual circumstances. This suggests a dual role for human planners, who should oversee the technical aspects and provide emotional and contextual insights that AI cannot currently offer [50, 51]. Such hybrid models have been proposed to safeguard relational and ethical aspects of care planning while leveraging AI’s strengths [34].

The participants also recognise technical challenges and the dependency on accurate, up-to-date data. The efficiency and effectiveness of AI systems are highly dependent on the quality of the input data, and any inaccuracies or outdated information can lead to suboptimal scheduling decisions. This finding emphasizes the need for continuous data monitoring and updates to maintain AI performance [52].

Interestingly, while participants desired a technology that matches their preferences and outputs a schedule, there seems to be limited awareness of AI’s potential to dynamically adapt to changing requirements and integrate additional decision-making information. This highlights a potential gap in understanding AI’s capabilities, particularly in terms of its adaptability and ability to integrate dynamic decision-making. Beyond technical functionality, the acceptance of AI tools is deeply rooted in nurses’ perceptions of professional autonomy, trust, and alignment with core values. If AI is perceived as undermining professional judgment or reducing opportunities for individualized decision-making, it may face resistance even if technically efficient. Trust in AI is not only about system accuracy but also about transparency, explainability, and alignment with professional norms [33, 53–55]. As emphasized in recent studies, fostering this trust requires meaningful involvement of nurses in AI design and implementation, alongside targeted education on how AI systems function and where their limitations lie [35]. Strengthening nurses’ autonomy and control over how AI is used is thus vital to ensure that these tools support rather than constrain professional practice and ethical care.

Thus, it has been argued that training in AI is essential for nurses to take a leading role in technological transformation rather than remaining passive users, which supports the call for inclusive and iterative development strategies [56]. As they gain experience with future AI scheduling systems, their requirements and expectations will likely evolve. Therefore, the development of such solutions necessitates an iterative process. This involves collecting feedback based on real-world interactions with the tool and continuously optimizing the system to align with users’ needs and workflows. This approach ensures that the AI system remains relevant, usable, and effective over time.

However, before organizations can benefit from AI’s potential, several systemic and organizational preconditions must be met. Studies show that the level of AI hype has significantly outpaced its scientific maturity and practical validation, especially in clinical settings [57, 58]. Healthcare managers often encounter barriers such as insufficient leadership readiness, limited financial and human resources, and time constraints due to workforce shortages [59]. Moreover, past experiences with digital technologies, such as electronic health records, have revealed unintended consequences such as increased administrative burden, stress, and reduced job satisfaction among health professionals [60–64]. These negative outcomes are not only due to software complexity but also hardware limitations, like outdated systems or frequent technical errors [65]. Such experiences contribute to technostress, defined as the discomfort or anxiety individuals feel when using or learning new technologies [66]. This phenomenon can foster resistance toward future digital innovations, including AI [67–69].

Therefore, careful planning and assessment of organizational readiness are critical. Organizational readiness for AI is a multi-dimensional concept encompassing financial and human resources, stable IT infrastructure, clear strategic alignment, participatory decision-making, and staff competencies and motivation [70–73]. Without these conditions, implementation risks failure and may exacerbate stress rather than alleviate it. Ensuring an ethically grounded, inclusive, and context-sensitive approach to AI adoption is thus essential.

### Strengths and limitations

The study used FG and knowledge mapping to collect perspectives and ensure a structured and transparent analysis. The diversity of the sample and the use of interview guidelines encouraged dynamic discussions. This study used focus groups and knowledge mapping to collect diverse perspectives and ensure a structured and transparent analysis. The combination of interactive group discussion and visual real-time mapping represents a novel and participatory methodological approach that enabled participants to co-construct and reflect upon key themes. The inclusion of participants from different professional roles nurse leaders, permanent staff, and temporary nurses allowed for a multi-perspective understanding of fairness, transparency, and scheduling challenges. Furthermore, the study addresses a highly relevant and under-researched topic with direct implications for future developments in AI-based workforce management. However, several limitations must be considered.

First, transferability is limited due to the convenience sampling and the online implementation. The choice of online FG, while practical, may have excluded individuals with limited digital access or confidence, introducing potential selection bias. Furthermore, conducting sessions via Microsoft Teams may have limited the depth of interaction, reduced non-verbal communication cues, and potentially affected the richness of the data compared to face-to-face FG.

Second, using FG rather than structured group interviews aimed to foster interaction among participants but may have also introduced group dynamics effects. For instance, dominant participants (“power talkers”) may have disproportionately shaped the discussions, influencing the data collected.

Third, the real-time visualization of discussion points through knowledge mapping provided structure and transparency but may have simultaneously guided participant focus, constrained exploration of emerging ideas, and simplified complex perspectives into predefined clusters.

Fourth, the recordings were not fully transcribed and analyzed; instead, the knowledge maps and notes served as the primary data sources. This methodological choice may have led to the omission of subtle nuances in participant statements, thus diminishing the interpretative depth of the findings.

Lastly, the interview guides and the explanations of AI provided to participants could have framed their understanding and expectations of AI-supported duty scheduling, introducing potential bias in how they articulated its possible roles and challenges. However, the use of mind mapping as a method for data analysis does not appear to yield inferior results compared to thematic analysis, as shown in prior research [41], supporting the validity of the findings despite the chosen analytical approach.

### Implications for practice

The findings of this study have several practical implications for healthcare organizations implementing AI-based shift-planning systems.

First, it is important that nurses are actively involved in the planning and implementation phases to ensure that AI-driven systems align with their needs and preferences. The study findings emphasize that participatory approaches can enhance acceptance and usability. Through targeted training and ongoing support, nurses can familiarize themselves with new technology and integrate it smoothly into their workflow. This co-creation process should include feedback mechanisms, involvement in pilot phases, and structured dialogue between developers and users.

Moreover, ensuring that AI systems are regularly updated with accurate data is vital to maintaining reliability. Participants also underscored the need for transparent communication about how AI-based scheduling works, including its limitations and decision-making processes. Providing clear information and training on the functionality and potential applications of AI-based shift planning can help foster trust and understanding among healthcare staff. Concrete measures might include regular staff workshops, quick-reference guides, and a dedicated support team for troubleshooting and onboarding. This will also facilitate an awareness of how technological efficiency can complement, rather than replace, the human aspects of workforce planning.

### Future research

Further research should investigate the long-term effects of integrating AI into shift scheduling, specifically regarding WLB, job satisfaction, and employee retention. Future studies should also explore which specific features of AI systems most influence perceptions of fairness and transparency and the most effective strategies for involving staff in their development and implementation.

Additionally, it is important to examine the psychological impact of AI-supported scheduling, including potential stress or relief experienced by employees. More research is needed to better understand the underlying needs of scheduling itself, what AI should ideally achieve, and whether alternative approaches might be more effective. This includes exploring how human-AI collaboration can be designed to foster mutual trust and support employee well-being.

## Conclusions

This study highlights the perceptions of current scheduling systems and the perceived potential of AI-based systems to improve WLB, job satisfaction, and fairness within the nursing profession. Although challenges and limitations exist, the benefits of increased accuracy, efficiency, and transparency may be significant. By involving nurse professionals in the development process and maintaining a balance between technology and human oversight, AI can be a powerful tool for optimising shift scheduling in healthcare settings. This reinforces the importance of co-creation and interdisciplinary collaboration in future AI implementations.

## Supplementary Information

Below is the link to the electronic supplementary material.

Supplementary Material 1

Supplementary Material 2

Supplementary Material 3

Supplementary Material 4

Supplementary Material 5

## Data Availability

The interview data analysed in the current study are available to the corresponding author on reasoned request.
