# Supplementary material for: Exploring nurse perspectives on AI-based shift scheduling for fairness, transparency, and work-life balance
Source: BMC Nurs. 2025 Sep 2;24:1161. doi: 10.1186/s12912-025-03808-0 (PMC12406402; doi:10.1186/s12912-025-03808-0)
Supplement: Supplementary file 3 — Supplementary Material 3 [file 12912_2025_3808_MOESM3_ESM.docx]

**AI-based duty rostering project**

**Interview guide for those affected - temporary employees**

**Introduction Conversation:**

- Thank you for your willingness to take part in the interview.
- Aim: Today's interview is part of a feasibility study to evaluate the views and expectations of AI-based duty scheduling. This should be fair and respond to the individual wishes of the employees. The aim of this study is to develop duty scheduling that is fair for the company and the employee and to improve the health, satisfaction and resilience of employees. In addition, AI-based duty scheduling should reduce the conflict between work and family.

The specific aim of today's interview is to find out whether you see a fundamental need for AI-based duty scheduling. You can share your requirements, expectations and challenges.

Your data mainly contributes to the decision-making basis for analysing the potential for a follow-up project. Your data can therefore be incorporated into the preparation of an application for a follow-up project.

- Time frame of the interview: approx. 90 minutes
- Check declarations of consent
- Obtain consent for recording the conversation
- Clarify open questions
- Start call recording

| **Entry phase** | | |
| --- | --- | --- |
| 1. **How do you experience your current rota planning?** | | |
| **Subject area:**   - Recording the current situation | **Concrete enquiries:**   - How do you plan your services? - Why did you decide to work on a temporary basis? - How satisfied are you with the duty rostering process and why? - What advantages and disadvantages do you see in your duty rota? - What influence does work organisation have on your work-life balance? - Where do you see the advantages and disadvantages of an 8- and 12-hour shift?   (12-hour shifts: max. 4 days in a row, at 100% approx. 14 days off, shift from 7:00-19:30, two handover reports/day)   - What would have to change for you to be permanently employed again? | **Checklist:**   - Current planning - Reason for working temporarily - Satisfaction - Effects on work-life balance |

| **Main phase**  **Description of AI-based duty scheduling**  As mentioned at the beginning, AI-based duty scheduling should lead to staff preferences and needs being taken into account more effectively. Employees should receive a more balanced and fair roster that minimises fatigue caused by shift changes and enables an improved work-life balance. The needs of employees should be better met by adapting to individual shift preferences and taking shift compositions into account. The participation of employees in shift planning should increase their satisfaction and work motivation.  AI-based duty planning makes it easier for planners to write the duty rota within a reasonable period of time and to make it transparent how decisions are made. By utilising AI algorithms, the tool can also support the search for replacements by drawing on stored data and identifying suitable candidates for additional assignments. AI-based duty scheduling means that managers have more time to manage their employees. |
| --- |

| 1. **What does fair and participatory duty scheduling mean to you?** | | |
| --- | --- | --- |
| **Subject area:**   - Fair and participative | **Concrete enquiries:**   - What do you mean by fair duty rostering? - What does participatory duty scheduling mean to you? | **Checklist:**   - Gaining a "fair and participatory" understanding of spas |
| 1. **What requirements do you have in terms of duty scheduling to ensure a good work-life balance?** | | |
| **Subject area:**   - Requirements for duty scheduling | **Concrete enquiries:**   - What factors do you see influencing your work-life balance in relation to your duty rota? - Which shifts or shift patterns do you prefer for a satisfactory work-life balance? - Which shifts or shift patterns do you think have a negative impact on work-life balance? - Does controlling your duty rota have an impact on your work-life balance? - What challenges do you see in dual roles such as childcare? - Are you more flexible as a temporary carer than as a permanent carer? | **Checklist:**   - Influencing factors WLB - Requirements |
| 1. **What expectations do you have of AI-based, fair and participatory duty scheduling?** | | |
| **Subject area:**   - Description and recording of expectations & requirements | **Concrete enquiries:**   - What expectations would have to be met for AI-based duty rostering to improve your rota? - Do you have fundamental trust in an artificial intelligence that creates your rota? - What would strengthen or weaken your trust? - What functions would you like to see in AI-based duty rostering to improve your work-life balance? - *What would an AI-based duty roster tool have to include for you to use it?* | **Checklist:**   - General expectations - Trust - AI and WLB |
| 1. **Where do you see possible advantages and disadvantages of AI-based duty rostering?** | | |
| **Subject area:**   - Hurdles/challenges | **Concrete enquiries:**   - What advantages or opportunities do you see in fair and participatory AI-based duty scheduling? - What difficulties or disadvantages do you see in fair and participatory AI-based duty rostering? - What advantages / disadvantages of AI-based duty scheduling do you see in terms of work-life balance? | **Checklist:**   - Advantages / disadvantages - WLB |
| **Finalisation phase** | | |
| - **We are approaching the end of the interview. Is there anything else you would like to say about this topic that you think is important?** - **Would you be interested in participating in the project to develop AI-based duty rostering?**   **Conclusion:**   - Do you have any further questions for us? - Thanking and saying goodbye - Stop recording | | |

**References**

1. Al‐Hammouri MM, Rababah JA. Work family conflict, family work conflicts and work‐related quality of life: The effect of rotating versus fixed shifts. J Clin Nurs John Wiley Sons Inc. August 2023;32(15/16):4887–93.

2. Anderson L. The impact of paramedic shift work on the family system: a literature review. Br Paramed J. 1. März 2019;3(4):43.

3. Baljani E, Rezaee Moradali M, Hajiabadi NR. The nurse manager support process in the work–family conflict of clinical nurses: a qualitative study. J Res Nurs. November 2023;28(6/7):499–513.

4. Barnes-Farrell JL, Laguerre RA, Di Milia LV. An evaluation of morningness and schedule misfit using the revised Preferences Scale (PS-6): Implications for work and health outcomes among healthcare workers. Chronobiol Int. Mai 2023;40(5):612–25.

5. Beyramijam M, Akbari Shahrestanaki Y, Khankeh H, Aminizadeh M, Dehghani A, Hosseini MA. Work-Family Conflict among Iranian Emergency Medical Technicians and Its Relationship with Time Management Skills: A Descriptive Study. Emerg Med Int. 2020;2020:7452697.

6. Chen S, Wu H, Sun M, Wei Q, Zhang Q. Effects of shift work schedules, compensatory sleep, and work–family conflict on fatigue of shift‐working nurses in Chinese intensive care units. Nurs Crit Care. November 2023;28(6):948–56.

7. Dhaini SR, Denhaerynck K, Bachnick S, Schwendimann R, Schubert M, De Geest S, u. a. Work schedule flexibility is associated with emotional exhaustion among registered nurses in Swiss hospitals: A cross-sectional study. Int J Nurs Stud. Juni 2018;82:99–105.

8. Elsie Eunice Amoo Asiedu, Annor F, Kwesi Amponsah‐Tawiah, Kwasi Dartey‐Baah. Juggling family and professional caring: Role demands, work–family conflict and burnout among registered nurses in Ghana. Nurs Open. Oktober 2018;5(4):611–20.

9. Emmanuel T, Griffiths P, Lamas-Fernandez C, Ejebu O, Dall’Ora C. The important factors nurses consider when choosing shift patterns: A cross-sectional study. J Clin Nurs. 27. Dezember 2023;

10. Goong H, Xu L, Li C‐ yu. Effects of work-family-school role conflicts and role-related social support on burnout in Registered Nurses: a structural equation modelling approach. J Adv Nurs John Wiley Sons Inc. November 2016;72(11):2762–72.

11. Han S, Kwak S. The effect of sleep disturbance on the association between work–family conflict and burnout in nurses: a cross-sectional study from South Korea. BMC Nurs. 2022;21:1–8.

12. Hauser C, Stahl J, Simon M, Valenta S, Favez L, Zúñiga F. Identifying work‐related factors associated with work–family conflict of care workers in nursing homes: A cross‐sectional study. J Adv Nurs John Wiley Sons Inc. Oktober 2023;79(10):3935–45.

13. Jennings K, Sinclair R, Mohr C. Who Benefits From Family Support? Work Schedule and Family Differences. J Occup Health Psychol. Januar 2016;21(1):51–64.

14. Kunst J, Loset G, Hosoy D, Bjorvatn B, Moen B, Mageroy N, u. a. THE RELATIONSHIP BETWEEN SHIFT WORK SCHEDULES AND SPILLOVER IN A SAMPLE OF NURSES. Int J Occup Saf Ergon. 2014;20(1):139–47.

15. Lembrechts L, Dekocker V, Zanoni P, Pulignano V. A study of the determinants of work-to-family conflict among hospital nurses in Belgium. J Nurs Manag. Oktober 2015;23(7):898–909.

16. Mauno S, Ruokolainen M, Kinnunen U. Work-family conflict and enrichment from the perspective of psychosocial resources: comparing Finnish healthcare workers by working schedules. Appl Ergon. Mai 2015;48:86–94.

17. Naidu P, Buccimazza I. Surgery in South Africa - challenges and barriers. South Afr J Surg Suid-Afr Tydskr Vir Chir. September 2021;59(3):77–81.

18. Navarro Moya P, Villar Hoz E, González Carrasco M. How medical transport service professionals perceive risk/protective factors with regard to occupational burnout syndrome: Differences and similarities between an Anglo-American and Franco-German model. Work Read Mass. 2020;67(2):295–312.

19. Oh HK, Cho SH. Effects of nurses’ shiftwork characteristics and aspects of private life on work-life conflict. PLOS ONE. 1. Dezember 2020;15(12):e0242379.

20. Peters V, Houkes I, de Rijk A, Bohle P, Engels J, Nijhuis F. Which resources moderate the effects of demanding work schedules on nurses working in residential elder care? A longitudinal study. Int J Nurs Stud. Juni 2016;58:31–46.

21. Pisarski A, Barbour J. What roles do team climate, roster control, and work life conflict play in shiftworkers’ fatigue longitudinally? Appl Ergon. Mai 2014;45(3):773–9.

22. Sarıtaş Canet Tuba. Precarious contours of work–family conflict: The case of nurses in Turkey. Econ Labour Relat Rev ELRR. März 2020;31(1):59–75.

23. Skoufi GI, Lialios GA, Papakosta S, Constantinidis TC, Galanis P, Nena E. Shift Work and Quality of Personal, Professional, and Family Life among Health Care Workers in a Rehabilitation Center in Greece. Indian J Occup Environ Med. September 2017;21(3):115–20.

24. Suter J, Kowalski T. The impact of extended shifts on strain-based work-life conflict: A qualitative analysis of the role of context on temporal processes of retroactive and anticipatory spillover. Hum Resour Manag J. April 2021;31(2):514–31.

25. Uhde A, Schlicker N, Wallach DP, Hassenzahl M. Fairness and Decision-making in Collaborative Shift Scheduling Systems. Proc 2020 CHI Conf Hum Factors Comput Syst. 21. April 2020;1–13.

26. Booker LA, Fitzgerald J, Mills J, Bish M, Spong J, Deacon-Crouch M, u. a. Sleep and fatigue management strategies: How nurses, midwives and paramedics cope with their shift work schedules-a qualitative study. Nurs Open. Januar 2024;11(1):e2099.

27. Zhang Y, Punnett L, Nannini A. Work-Family Conflict, Sleep, and Mental Health of Nursing Assistants Working in Nursing Homes. Workplace Health Saf. Juli 2017;65(7):295–303.

28. Alharbi MF, Alahmadi, BA, Alali, M, Alsaedi S. Quality of Nursing Work Life Among Hospital Nurses in Saudi Arabia: a Cross-Sectional Study. J Nurs Manag. 2019;27(8):1722‐1730.

29. Elinich J, Wynarczuk KD, Mccormick E. Perceptions and experiences of burnout: A survey of physical therapists across practice settings and patient populations. Physiother THEORY Pract. 14. Oktober 2023;

30. Haller T, Quatrara B, Miller-Davis C, Noguera A, Pannone A, Keim-Malpass J, u. a. Exploring Perceptions of Shift Length: A State-Based Survey of Registered Nurses. J Nurs Adm. September 2020;50(9):449–55.

31. Konkol M, George EL, Scott PW, Imes CC. Examining Nurses’ Perception of Shift Work and Evaluating Supportive Interventions. J Nurs CARE Qual. März 2024;39(1):10–7.

32. Lauchart M, Ascher P, Kesel K, Weber S, Grabein B, Schneeweiss B, u. a. Compatibility of Work and Family Life: Survey of Physicians in the Munich Metropolitan Area. GESUNDHEITSWESEN. April 2019;81(4):299–308.

33. Lederer W, Paal P, von Langen D, Sanwald A, Traweger C, Kinzl JF. Consolidation of working hours and work-life balance in anaesthesiologists - A cross-sectional national survey. PLOS ONE. 31. Oktober 2018;13(10).

34. Lee H, Burrows HL, Singer K, Brower KJ, Bradford CR, Spencley B, u. a. Operational Constraints and Gender Biases: A Qualitative Analysis of Physician Parenting Experiences. WOMENS Health Rep. 2022;3(1):297–306.

35. Moore LR, Ziegler C, Hessler A, Singhal D, LaFaver K. Burnout and Career Satisfaction in Women Neurologists in the United States. J WOMENS Health. 1. April 2019;28(4):515–25.

36. Obina WF, Ndibazza J, Kabanda R, Musana J, Nanyingi M. Factors associated with perceived work-life balance among health workers in Gulu District, Northern Uganda: a health facility-based cross-sectional study. BMC PUBLIC Health. 23. Januar 2024;24(1).

37. Ogeill RP, Savic M, Ferguson N, Lubman D I. Shift-Work-Play: Understanding the positive and negative experiences of male and female shift workers to inform opportunities for intervention to improve health and wellbeing. Aust J Adv Nurs. Mai 2021;38(2):23–33.

38. Shiffer D, Minonzio M, Dipaola F, Bertola M, Zamuner AR, Dalla Vecchia LA, u. a. Effects of Clockwise and Counterclockwise Job Shift Work Rotation on Sleep and Work-Life Balance on Hospital Nurses. Int J Environ Res Public Health. September 2018;15(9).

39. Slusser K, Knobf MT, Linsky S, Kaisen A, Parkosewich J, Sterne P, u. a. A Focus Group Study of Retirement-Age Nurses: Balancing Tension and a Love of Nursing in a Changing Healthcare Environment. J Nurs Adm. Dezember 2022;52(12):646–52.

40. Stimpfel AW, Arabadjian M, Liang E, Sheikhzadeh A, Weiner SS, Dickson VV. Organization of Work Factors Associated with Work Ability among Aging Nurses. West J Nurs Res. Juni 2020;42(6):397–404.

41. Turk M, Davas A, Tanik FA, Montgomery AJ. Organizational stressors, work-family interface and the role of gender in the hospital: Experiences from Turkey. Br J Health Psychol. Mai 2014;19(2):442–58.

42. West S, Rudge T, Mapedzahama V. Conceptualizing nurses’ night work: an inductive content analysis: Journal of Advanced Nursing (John Wiley & Sons, Inc.). J Adv Nurs John Wiley Sons Inc. August 2016;72(8):1899–914.

43. Wynendaele H, Gemmel P, Pattyn E, Myny D, Trybou J. Systematic review: What is the impact of self-scheduling on the patient, nurse and organization? J Adv Nurs. Januar 2021;77(1):47–82.
