# Supplementary material for: Exploring nurse perspectives on AI-based shift scheduling for fairness, transparency, and work-life balance
Source: BMC Nurs. 2025 Sep 2;24:1161. doi: 10.1186/s12912-025-03808-0 (PMC12406402; doi:10.1186/s12912-025-03808-0)
Supplement: Supplementary file 4 — Supplementary Material 4 [file 12912_2025_3808_MOESM4_ESM.docx]

Supplementary material 4

Main responses of the key themes

| Main topics | Key responses |
| --- | --- |
| Experiencing current Work Scheduling | - Work scheduling planning is the most important operational task and requires structured, safe planning.  - Work scheduling is dependent on the company structure, safety, and standards.  - Monthly Work scheduling planning involves many overtime hours and the need for flexibility.  - Planning for the future is difficult due to the high number of overtime hours and gaps in Work scheduling.  - Due to the scheduling, staff is regularly required to work beyond their scheduled hours.  - Coordination of duties must be transparent.  - Adequate preparation is needed for shifts.  - The aim is to ensure a fair distribution of shifts and rest periods.  - High flexibility is essential.  - Employees must be involved in the dialogue.  - Fixed shift plans do not exist.  - There should be no 12-hour shifts.  - Rest periods should be respected.  - Special requests should be considered.  - Transparent communication between teams and shifts is necessary.  - No 12-hour shifts should be imposed.  - A clear and transparent communication strategy is required for efficient a high number of planning. |
| Requirements for Work Scheduling | - Must include self-service in Work scheduling planning. - Shifts must last 2.5 to 3 days. - Minimize back-and-forth in planning. - Less flexibility required for routine tasks. - Employees are to be granted flexibility for private events. - Younger employees require more flexibility, while older employees require less. - Early planning (4-6 weeks in advance) is important. - Hospitalization should only occur in exceptional cases. - Continuity of duty is essential. - Childcare-friendly duties. - Satisfaction improves when employees can select shifts. - If there is enough rest after shifts, shifts should start earlier.  - Increase day shifts with fewer evening and weekend shifts. - Prefer shifts at other locations or stations. - Shift planning should consider the mix of full-time and part-time employees. - Older employees wish to retain benefits for a longer period. - Utilization of Work-Life Balance to offer flexibility. - Retain employees by allowing them to work for another 1-2 years, beyond the typical retirement age. - Flexible attendance is important. - Approval of services during holidays and free periods. - Consideration of employees' preferences and strengths. - Reduction in the number of overtime and additional shifts. - Balance between work and life must be ensured by considering temporary employment. - Work-Life Balance should be utilized for older employees as well. - Participation in a high number of planning must be possible. - Hobbies should be taken into account. - Employees should have the option to waive the 12-hour shifts if they wish. - Extended model: 12-hour shifts followed by 8-hour night shifts to ensure better continuity. - Flexibility in changing workstations should be considered. - Better consideration of employees with children. - Part-time work should be possible. - Higher flexibility desired for different shifts.  - Introduction of a flexible Work scheduling. - More flexibility, less rigidity in a high number of planning. |
| Fair and Participatory Work Scheduling | - Fair distribution of shifts (early, late, night). - Shift preferences should be communicated. - Employees should be able to choose their shifts. - Equal distribution of weekend shifts is required. - Shifts should be communicated in a timely manner to avoid overtime. - Equalization of weekend shifts and consideration of individual employee needs. - Employees should be involved in planning and have equal opportunities for feedback. - A maximum of two weekend shifts per month should be allowed. - Ensuring a balanced workload is crucial for fairness. - Employees should have flexibility and the ability to influence the planning process. - Transparent planning is essential. - Participation in a high number of planning should be possible for all employees. - Transparent communication is key to a fair planning process. - Shift requests should be considered to improve fairness. |
| Requirements for AI in Work Scheduling | - Fairness and equal distribution should be provided by AI.  - Employees should have the ability to influence the planning process.  - AI should ensure an objective, non-prejudiced distribution of shifts.  - Shift planning must be transparent and free of any discrimination.  - Communication between teams should be improved through AI.  - AI must ensure that weekend shifts are distributed fairly.  - AI should respect employees' preferences and adjust the schedule accordingly.  - AI should improve the planning process by ensuring shifts are allocated fairly.  - AI should support transparent communication and better cooperation between teams.  - AI must ensure that overtime is minimized.  - AI should help balance employees' work and life through better planning.  - AI should allow for more individualized planning,  taking into account personal preferences.  AI must support transparent communication to improve overall team morale.  - AI should ensure fairness in the planning process.  - AI should also consider employees' strengths and assign tasks accordingly.  - AI must provide transparency and objectivity in Work scheduling planning. |
| Advantages and Disadvantages of AI-Based Work Scheduling | Pros:  - Better planning with AI. - Better alignment of shifts to employees' strengths. - Reduction of errors in planning. - AI can create more balanced Work scheduling. - Easier shift changes and less manual effort. - AI provides transparency. - Better recognition of employees' needs. - AI offers precise planning with clear communication.  - AI supports employee satisfaction through fair shift planning.  Cons: - AI cannot replace the human aspect of planning. - Some employees may be resistant to using AI. - Potential for mistrust if AI decisions are not well-explained. - AI requires training and data to function correctly. - AI must be monitored to ensure fairness. - AI may not fully understand individual employee needs. - Manual adjustments may still be necessary to accommodate unique situations. - AI cannot account for spontaneous changes or emergencies. - AI should not replace human interaction in the planning process.  - Data privacy concerns related to AI usage |
