# Supplementary figures and images for: Exploring nurse perspectives on AI-based shift scheduling for fairness, transparency, and work-life balance
Source: BMC Nurs. 2025 Sep 2;24:1161. doi: 10.1186/s12912-025-03808-0 (PMC12406402; doi:10.1186/s12912-025-03808-0)

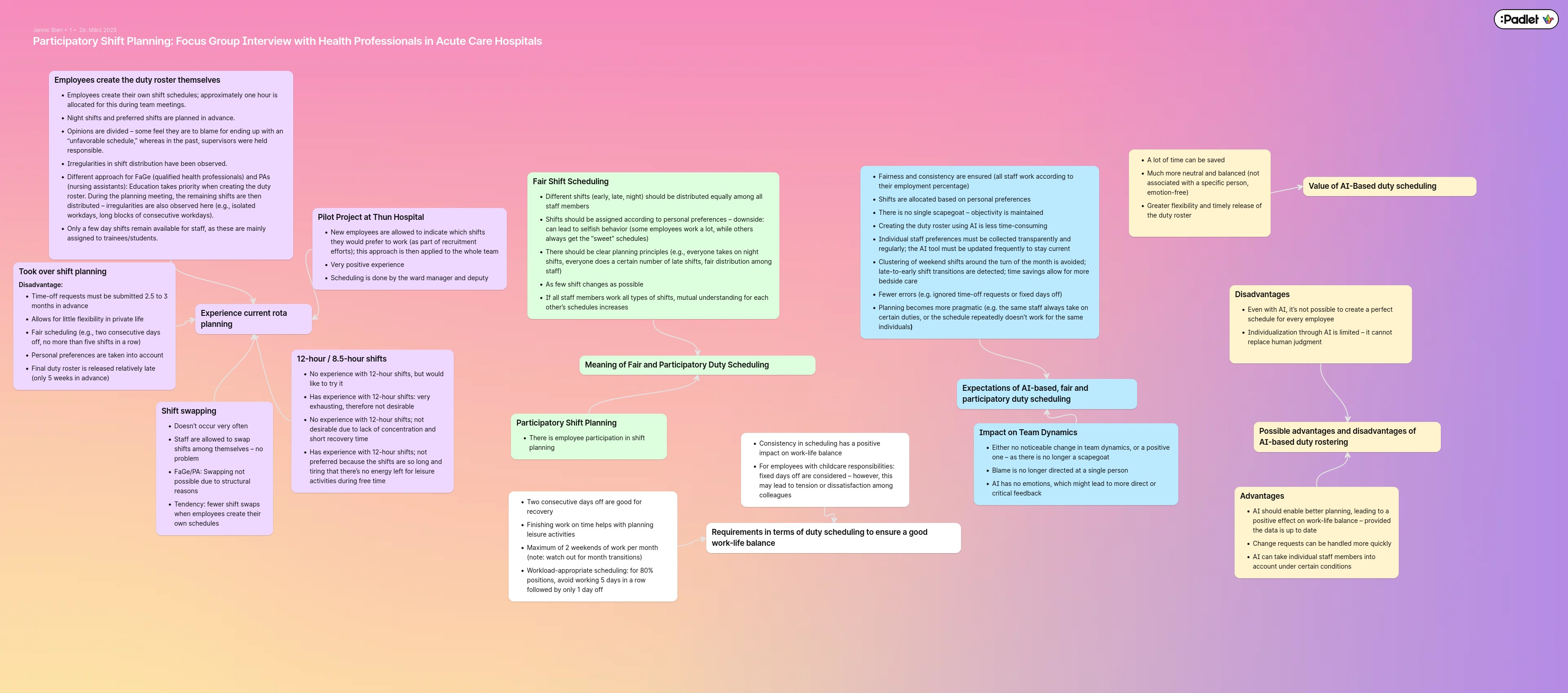

Supplement: Supplementary file 5 — Supplementary Material 5 [file 12912_2025_3808_MOESM5_ESM.png]
